# Evaluation of the COVID-19 Pandemic Intervention Strategies with Hesitant F-AHP

**DOI:** 10.1155/2020/8835258

**Published:** 2020-08-20

**Authors:** Funda Samanlioglu, Burak Erkan Kaya

**Affiliations:** Department of Industrial Engineering, Kadir Has University, Cibali 34083, Istanbul, Turkey

## Abstract

In this study, a hesitant fuzzy AHP method is presented to help decision makers (DMs), especially policymakers, governors, and physicians, evaluate the importance of intervention strategy alternatives applied by various countries for the COVID-19 pandemic. In this research, a hesitant fuzzy multicriteria decision making (MCDM) method, hesitant fuzzy Analytic Hierarchy Process (hesitant F-AHP), is implemented to make pairwise comparison of COVID-19 country-level intervention strategies applied by various countries and determine relative importance scores. An illustrative study is presented where fifteen intervention strategies applied by various countries in the world during the COVID-19 pandemic are evaluated by seven physicians (a professor of infectious diseases and clinical microbiology, an infectious disease physician, a clinical microbiology physician, two internal medicine physicians, an anesthesiology and reanimation physician, and a family physician) in Turkey who act as DMs in the process.

## 1. Introduction

As was realized from the previous 2009 AH1N1 pandemic and the recent COVID-19 pandemic, countries need efficient mitigation planning to prevent mass infection and fatalities. An effective intervention plan may help flatten the epidemic curve and, with protective measures, there might be a delay and reduction in the peak of the outbreak. As a result, the number of cases at any time stays under the surge capacity of a country's healthcare system. If the surge capacity of a country's healthcare system is exceeded, the morbidity and mortality rates increase for all hospitalized patients, not just for COVID-19 cases.

The basic reproduction number (*R*_0_) is the key factor that shows the strength of the epidemic; it is, without any interventions, the mean number of secondary cases generated by a single infected case in a population with no immunity to infection [1]. When *R*_0_ is greater than 1, the epidemic takes hold, and the overall fraction of population likely to be infected without interventions is the area under the epidemic curve which can be calculated roughly with 1-1/*R*_0_ [2]. Effective intervention strategies, if followed properly, might reduce the *R*_0_ below 1 and control the spread of COVID-19.

Countries need a systematic approach to determine which intervention strategies to apply during the COVID-19 pandemic and future potential epidemiological waves and pandemics. In this study, intervention strategies applied by countries during the COVID-19 pandemic are evaluated in terms of importance with the help of an MCDM method, hesitant F-AHP. Scenarios for the potential spread and impact of COVID-19 in the EU/EEA, with suggested actions for containment, were given in Johnson et al.'s article [3]. Also, Cheng et al. [4] presented an extensive dataset of government responses to the COVID-19 pandemic, and the interventions that are evaluated in this study are taken from their research. A list of these interventions is presented in Table 1 with detailed explanations and country examples.

In the literature, there is a limited number of studies that focus on the evaluation of intervention strategies. Aledort et al. [5] evaluated the evidence base for nonpharmaceutical public health interventions in an influenza pandemic with the help of literature review and expert opinions. Ciofi degli Atti et al. [6] evaluated the diffusion of pandemic influenza in Italy and the impact of various control measures with the help of SEIR (Susceptible-Exposed-Infected-Recovered) and individual-based models. Ajelli et al. [7] presented the real-time modeling analysis to estimate the impact of the pandemic and the mitigation measures during the 2009 A/H1N1v pandemic in Italy. Kohlhoff et al. [8] carried out an observational study and evaluated hospital mass screening and infection control practices with a pandemic influenza full-scale exercise in three acute care hospitals in Brooklyn, NY. Ventresca and Aleman [9] investigated the effects of six vaccination strategies in terms of the ability to contain disease spread by constructing a representative social network from the census of the Greater Toronto Area. Schiavo et al. [10] presented a review about evidence on interventions to communicate risk and promote disease mitigation measures in epidemics. Russell et al. [11] conducted a household survey in a school district of Kentucky to evaluate the effect of school closure mitigation on the transmission of influenza-like illness. Luca et al. [12] developed a stochastic spatial age-specific metapopulational model to investigate the impact of school closure on seasonal influenza epidemics in Belgium. Nicolaides [13] modified the SIR (Susceptible-Infected-Recovered) epidemic model to reflect the effects of hand washing in the infection process and investigated the effect of hand-hygiene mitigation strategy at airports for flu-type viruses.

MCDM methods have been rarely utilized to evaluate interventions. Shin et al. [14] used AHP to decide if private clinics and hospitals or public health centers should offer free vaccination to children in Korea. Mourits et al. [15] implemented EVAMIX (evaluations with mixed data) to rank control strategies for classical swine fever epidemics in the EU. Aenishaenslin et al. [16] implemented D-Sight (PROMETHEE with GAIA) to evaluate prevention and control strategies for the Lyme disease in Quebec, Canada. Pooripussarakul et al. [17] applied best-worst scaling to evaluate vaccines in Thailand. Previously, Samanlioglu [1] evaluated influenza intervention strategies in Turkey with fuzzy AHP-VIKOR.

In this study, various intervention strategies applied by countries in the world during the COVID-19 pandemic are evaluated by seven physicians with different expertise, acting as consultants and decision makers (DMs). For pairwise comparison of importance of strategies, as the MCDM method, hesitant F-AHP is applied. With (hesitant fuzzy) AHP, utilizing pairwise comparisons of alternatives and consistency check of these comparisons, dependable alternative scores can be determined. In this research, hesitant F-AHP is preferred over AHP or fuzzy AHP (F-AHP) since, different from AHP, with F-AHP, the uncertainty and vagueness on DMs' judgments can also be captured. Moreover, with the usage of multiple linguistic expressions and “hesitant” terminologies in hesitant F-AHP, more flexibility is attained in decision making than F-AHP since the degree of hesitation that DMs might have in reality can also be reflected.

## 2. Literature Review

In AHP [18], with its multilevel and hierarchical structure, alternatives are evaluated with respect to each criterion with pairwise comparisons and ranked based on a total weighted score. To reflect the obscurity and fuzziness of DMs' judgments, utilizing the concepts of fuzzy set theory [19, 20], F-AHP was developed and is used in many MCDM problems in the literature [21–24]. Fuzzy set theory contains classes with unsharp boundaries [25, 26], and crisp theory sets can be fuzzified by implementing the fuzzy set concepts [19]. In the literature, different extensions of fuzzy sets, such as intuitionistic fuzzy sets [27, 28], Pythagorean fuzzy sets [29–31], picture fuzzy sets [32–35], spherical fuzzy sets [36–43], and hesitant fuzzy sets [44–47], were used to deal with uncertainties in decision making problems.

In F-AHP, for pairwise comparisons, DMs give a single linguistic expression; and this does not reflect the hesitations DMs might have in reality. However, in hesitant F-AHP, DM might utilize hesitant fuzzy set (HFS) concepts [44, 45], hesitant fuzzy linguistic term sets (HFLTS) [44, 46], and multiple linguistic expressions and “hesitant” terminologies in their evaluations, which increase the flexibility and accuracy of the decision making process [47]. For example, while comparing interventions 1 and 8 pairwise, DM might give the following assessment: “intervention 1 is between absolutely strong and very strong in comparison to criterion 8”.

Hesitant F-AHP was implemented in several MCDM problems in the literature. Some of these applications are assessment of suppliers [48], cargo company performance [49], woodwork manufacturing CNC routers [50], sustainability of hydrogen production methods [51], summer sport schools [52], power generation enterprises [47], and innovation projects [53].

Until now, to the best of the authors' knowledge, hesitant F-AHP has never been studied for the evaluation of intervention strategies. With the proposed hesitant F-AHP, importance of countries' COVID-19 intervention strategies is systematically evaluated. Application steps of hesitant F-AHP are explained in Section 3. An illustrative study is given in Section 4 to demonstrate the implementation, along with the conclusion and discussion in Section 5.

## 3. Proposed Hesitant F-AHP Approach

In the proposed hesitant F-AHP, triangular fuzzy numbers (TFNs) are implemented due to their uncomplicatedness. A fuzzy number is a special fuzzy set *F*={(*x*, *μ*_*F*_(*x*)), *x* ∈ *R*}, where *R* : −*∞* < *x* < +*∞* and *μ*_*F*_(*x*) is from *R* to [0, 1]. A TFN, $\tilde{M}=\left(l,m,u\right)l\leq m\leq u$, has the triangular type membership function

(1) $$\begin{array}{c} \mu_{F}\left(x\right)=\left\{\begin{array}{cc} 0, & x<l,\\ \frac{x-l}{m-l}, & l\leq x\leq m,\\ \frac{u-x}{u-m}, & m\leq x\leq u,\\ 0, & x>u. \end{array}\right.. \end{array}$$

Arithmetic operations between two positive TFNs $\tilde{C}=\left(l_{1},m_{1},u_{1}\right)$, $\tilde{D}=\left(l_{2},m_{2},u_{2}\right)l_{1}\leq m_{1}\leq u_{1}l_{2}\leq m_{2}\leq u_{2}$ and a crisp number *E* are explained as [53–55]

(2) $$\begin{array}{c} \tilde{C}+\tilde{D}=\left(l_{1}+l_{2},m_{1}+m_{2},u_{1}+u_{2}\right),\\ \tilde{C}+E=\left(l_{1}+E,m_{1}+E,u_{1}+E\right),\\ \tilde{C}-\tilde{D}=\left(l_{1}-u_{2},m_{1}-m_{2},u_{1}-l_{2}\right),\\ \tilde{C}-E=\left(l_{1}-E,m_{1}-E,u_{1}-E\right),\\ \tilde{C}*\tilde{D}=\left(l_{1}*l_{2},m_{1}*m_{2},u_{1}*u_{2}\right),\\ \tilde{C}*E=\left(l_{1}*E,m_{1}*E,u_{1}*E\right),\;\text{for }E\geq 0,\\ \frac{\tilde{C}}{\tilde{D}}=\left(\frac{l_{1}}{u_{2}},\frac{m_{1}}{m_{2}},\frac{u_{1}}{l_{2}}\right),\\ \frac{\tilde{C}}{\tilde{D}}=\left(\min\left(\frac{l_{1}}{l_{2}},\frac{l_{1}}{u_{2}},\frac{u_{1}}{l_{2}},\frac{u_{1}}{u_{2}}\right),\frac{m_{1}}{m_{2}},\max\left(\frac{l_{1}}{l_{2}},\frac{l_{1}}{u_{2}},\frac{u_{1}}{l_{2}},\frac{u_{1}}{u_{2}}\right)\right), \end{array}$$

if $\tilde{C}$ and $\tilde{D}$ are two TFNs (not necessarily positive TFNs).

(3) $$\begin{array}{c} \frac{\overset{˜}{C}}{E}=\left(\frac{l_{1}}{E},\frac{m_{1}}{E},\frac{u_{1}}{E}\right),\;\text{for }E>0,\\ {\overset{˜}{C}}^{-1}=\left(\frac{1}{u_{1}},\frac{1}{m_{1}},\frac{1}{l_{1}}\right),\\ \text{MAX}\left(\overset{˜}{C}+\overset{˜}{D}\right)=\left(\max\left(l_{1},l_{2}\right),\max\left(m_{1},m_{2}\right),\max\left(u_{1},u_{2}\right)\right),\\ \text{MIN}\left(\overset{˜}{C}+\overset{˜}{D}\right)=\left(\min\left(l_{1},l_{2}\right),\min\left(m_{1},m_{2}\right),\min\left(u_{1},u_{2}\right)\right). \end{array}$$

For defuzzification of TFNs, “the graded mean integration approach” [56] is applied as

(4) $$\begin{array}{c} Crisp\left(\tilde{C}\right)=\frac{\left(4m_{1}+l_{1}+u_{1}\right)}{6}. \end{array}$$

In Triangular Fuzzy Hesitant Fuzzy Sets (TFHFS), the membership degree of an element to a given set is expressed by several possible TFNs. Several aggregation operators for TFHFS were introduced by Yu [57] for assessment of teaching quality.

If *X* is a fixed set, the HFS on *X* returns a subset of [0,1] by

(5) $$\begin{array}{c} G=\left\{\left.<x,h_{G}\left(x\right)>\right|x\in X\right\}, \end{array}$$

where *h*_*G*_(*x*) is the possible membership degrees of element *x* ∈ *X* to set *G* with values in [0,1]. The lower and upper bounds are calculated as

(6) $$\begin{array}{c} h_{\left(x\right)}^{-}=\min h\left(x\right),\\ h_{\left(x\right)}^{+}=\max h\left(x\right). \end{array}$$

Basic operations for 3 HFS, *h*, *h*1, *h*2, are given as

(7) $$\begin{array}{c} h^{\overset{‥}{u}}=\underset{\gamma \in h}{\cup^{}}\left\{\gamma^{\overset{‥}{u}}\right\},\\ \overset{‥}{u}h=\underset{\gamma \in h}{\cup^{}}\left\{1-{\left(1-\gamma \right)}^{\overset{‥}{u}}\right\},\\ h_{1}\pm h_{2}=\underset{\gamma 1\in h1,\gamma 2\in h2}{\cup^{}}\left\{\gamma^{1}+\gamma^{2}-\gamma^{1}\gamma^{2}\right\},\\ h_{1}\cap^{}h_{2}=\underset{\gamma 1\in h1,\gamma 2\in h2}{\cup^{}}\min\left\{\gamma^{1},\gamma^{2}\right\},\\ h_{1}\cup^{}h_{2}=\underset{\gamma 1\in h1,\gamma 2\in h2}{\cup^{}}\max\left\{\gamma^{1},\gamma^{2}\right\},\\ h_{1}⊗h_{2}=\underset{\gamma 1\in h1,\gamma 2\in h2}{\cup^{}}\left\{\gamma^{1}\gamma^{2}\right\}. \end{array}$$

“Ordered Weighting Averaging (OWA)” operator that can be employed is

(8) $$\begin{array}{c} \text{OWA}\left(a_{1},a_{2},\ldots ,a_{n}\right)=\sum_{j=1}^{n}w_{j}b_{j}, \end{array}$$

where *b*_*j*_ is the *j*th largest of *a*_1_, *a*_2_,…, *a*_*n*_, *w*_*j*_ ∈ [0,1]∀ *j*, and∑_*j*=1_^*n*^*w*_*j*_=1 [53, 58].

### 3.1. Fuzzy Envelope Approach in Hesitant F-AHP

“Fuzzy envelope approach” [59] is applied to combine DM evaluations in hesitant F-AHP. Scales given for DM evaluations are sorted from the lowest *s*_*o*_ to the highest *s*_*g*_, so if the DM's evaluations are between *s*_*i*_ and *s*_*j*_, then *s*_*o*_ ≤ *s*_*i*_ ≤ *s*_*j*_ ≤ *s*_*g*_.

Based on the HFLTS, linguistic expressions can be represented by a triangular fuzzy membership function $\tilde{A}=$ (*a*, *b*, *c*), where *a*, *b*, and *c* are calculated as

(9) $$\begin{array}{c} a=\min\left\{a_{L}^{i},a_{M}^{i},a_{M}^{i+1},\cdots ,a_{M}^{j},a_{R}^{j}\right\}=a_{L}^{i}, \end{array}$$

(10) $$\begin{array}{c} b=\left\{\begin{array}{c} a_{M}^{i}ifi+1=j,\\ {\text{OWA}}_{W}\left\{a_{M}^{i},a_{M}^{i+1},\cdots ,a_{M}^{j}\right\},\;\text{otherwise}, \end{array}\right. \end{array}$$

(11) $$\begin{array}{c} c=\max\left\{a_{L}^{i},a_{M}^{i},a_{M}^{i+1},\cdots ,a_{M}^{j},a_{R}^{j}\right\}=a_{R}^{j}. \end{array}$$

Weight vector in OWA operator [60] is defined as

(12) $$\begin{array}{c} w_{1}=\alpha^{n-1},w_{2}=\left(1-\alpha \right)\alpha^{n-2},\cdots ,w_{n}=\left(1-\alpha \right), \end{array}$$

where *α*=(*l* − *j*+*i*)/(*l* − 1).

Here, *l* depends on the number of terms in DM's evaluation scale (in Table 2), *j* is the rank of the highest, and *i* is the rank of the lowest evaluation value. *i* and *j* can take ranks starting from 0 to *l* and *n* = *j* − *i* [53, 58].

In the proposed hesitant F-AHP approach, the DMs make pairwise comparisons of importance of intervention strategy alternatives using the linguistic terms given in Table 2.

Steps of the proposed hesitant F-AHP are as follows:

 
*Step 1*. Identify *K* DMs, *n* alternatives (intervention strategies), and linguistic terms and scale for the pairwise comparison of alternatives. Each DM makes pairwise comparison of alternatives (intervention strategies) with respect to the importance criterion. Based on the scale used in Table 2 and utilizing equations (8)–(12), DM's assessments are combined with fuzzy envelope approach, and TFNs corresponding to the assessment of each DM are obtained. Calculate

(13) $$\begin{array}{c} {\tilde{x}}_{ij}=\frac{1}{K}\left({\tilde{x}}_{ij}^{1}\left(+\right){\tilde{x}}_{ij}^{2}\left(+\right)\cdots \left(+\right){\tilde{x}}_{ij}^{K}\right), \end{array}$$

  where ${\tilde{x}}_{ij}^{K}=\left(a_{ij}^{K},b_{ij}^{K},c_{ij}^{K}\right)\forall i,j,k$ is the TFN corresponding to the evaluation of the *K*th DM.
 
*Step 2*.

(14) $$\begin{array}{c} \tilde{X}=\left[\begin{array}{ccccc} \left(1,1,1\right) & {\tilde{x}}_{12} & .. & .. & {\tilde{x}}_{1n}\\ {\tilde{x}}_{21} & \left(1,1,1\right) & .. & .. & {\tilde{x}}_{2n}\\ .. & .. & .. & .. & ..\\ .. & .. & .. & .. & ..\\ {\tilde{x}}_{n1} & {\tilde{x}}_{n2} & .. & .. & \left(1,1,1\right) \end{array}\right], \end{array}$$

  with elements ${\tilde{x}}_{ij}=\left(a_{ij},b_{ij},c_{ij}\right)$ being then defuzzified with equation (4) and approximate alternative scores *w*=(*w*_1_, *w*_2_, ..., *w*_*n*_) being determined by averaging the entries on each row of normalized *X*. Therefore, the normalized principal eigenvector is *w*. The largest eigenvalue (principal eigenvalue, *λ*_max_) is determined with

(15) $$\begin{array}{c} Xw^{T}=\lambda_{\max}w^{T}. \end{array}$$

  The consistency index (*CI*) is

(16) $$\begin{array}{c} CI=\frac{\lambda_{\max}-n}{n-1}. \end{array}$$

  The consistency ratio (*CR*) is then used to assess the consistency of pairwise comparisons:

(17) $$\begin{array}{c} CR=\frac{CI}{RI}. \end{array}$$

 
*RI* is the random index, and if *CR* < 0.10, the comparisons are consistent and acceptable; otherwise, they are not [18].
 
*Step 3*. If comparisons are acceptable, rank the alternatives (intervention strategies) from the best to the worst based on approximate alternative scores *w*=(*w*_1_, *w*_2_, ⋯, *w*_*n*_) in decreasing order. Note that higher *w* shows better alternative.

## 4. Illustrative Study

In this study, 15 intervention strategies (A1, ⋯, A15) applied by countries worldwide during the COVID-19 pandemic are evaluated and compared in terms of the importance criterion by 7 physicians who act as DMs. In this study, DMs are a professor of infectious diseases and clinical microbiology (DM1), an infectious disease physician (DM2), a clinical microbiology physician (DM3), two internal medicine physicians (DM4 and DM5), a family physician (DM6), and an anesthesiology and reanimation physician (DM7) in Turkey.

In the proposed hesitant F-AHP, 7 DMs compare intervention strategy alternatives pairwise with the help of linguistic terms in Table 2, and the comparison is given in Table 3. After the combination of each DM's assessments with “fuzzy envelope approach” and aggregation of the corresponding TFNs of 7 DMs assessments, the fuzzy evaluation matrix for the alternative scores ($\tilde{X}$) in Table 4 is obtained.

Then, elements of *X* ˜ are defuzzified with equation (4), and evaluation matrix *X* in Table 5 is obtained. Afterwards, *w*=(*w*_1_, *w*_2_, ..., *w*_*n*_) is determined by taking the average of the entries on each row of normalized *X*. *λ*_max_= and CR of *X* is checked with equations (15)–(17) as *CI* = (16.268–15)/14 = 0.0906 and *CR* = *CI*/*RI* = 0.0906/1.59 = 0.05698. Since *CR* < 0.1, the pairwise comparisons are consistent and acceptable.

Based on the *w*=(*w*_1_, *w*_2_, ..., *w*_*n*_) obtained with hesitant F-AHP, intervention strategy alternatives are ranked in terms of importance criterion from the best to the worst as follows: declaration of emergency (A15), quarantine/lockdown of patients and those suspected of infection (A1), curfew (A13), common health testing (independent of suspected infection) (A12), social distancing (A3), closure of schools (A9), external border restrictions reducing the ability to exit or enter a country (A8), internal border restrictions reducing the ability to move freely (transportation) within a country (A2), restrictions of mass gatherings (A7), health monitoring (A4), restriction of nonessential government services (A14), government policies that affect the country's health resources (materials and health worker) (A10), formation of new task units/bureaus and government policies changing administrative capacity to respond to the crisis (A11), public awareness campaigns (A5), and restriction of nonessential businesses (A6).

## 5. Conclusion and Discussion

In this paper, a hesitant F-AHP approach is presented to help DMs such as policymakers, governors, and physicians evaluate and rank intervention strategy alternatives applied by various countries during the COVID-19 pandemic. At present, there does not appear to be a study in the literature that evaluates countries' COVID-19 intervention strategies. Moreover, in the literature a systematic MCDM approach such as hesitant F-AHP has never been utilized to evaluate and rank COVID-19 intervention strategies. Adoption of hesitant fuzzy linguistic terms in the process captures the fuzziness and hesitations and provides flexibility in decision making.

In the literature, AHP is mainly criticized due to the possible occurrence of rank reversal phenomenon caused by adding or deleting an alternative [61–64]. Adding a new alternative includes new information in the model, and therefore the decision needs to be reevaluated [65]. Unfortunately, the rank reversal problem occurs not only in AHP, but also in many other decision making approaches such as Borda–Kendall method, SAW, TOPSIS, and cross-efficiency evaluation method [61]. However, this limitation did not affect our analysis since we did not need to add or delete any new intervention alternatives.

For the illustrative study, expert opinion for the evaluations was needed, so a professor of infectious diseases and clinical microbiology, an infectious disease physician, a clinical microbiology physician, two internal medicine physicians, a family physician, and an anesthesiology and reanimation physician in Turkey acted as DMs. Based on their evaluation, declaration of emergency, quarantine/lockdown of patients and those suspected of infection, and curfew are determined as the best three intervention strategies among the evaluated ones.

In this research, intervention strategies are evaluated without taking into consideration the interventions' timing. In reality, the timing of the intervention, with respect to the beginning and peak of the epidemic, and duration of the application of the intervention are really significant. Therefore, when making decisions, DMs need to take those into consideration, as well as intervention strategy rankings. Based on these, the proposed hesitant F-AHP approach can be adopted and utilized by policy makers, governors, national public health departments, and physicians for the evaluation of countries' intervention strategies for COVID-19 and other future similar epidemics. Also, for future research, various other potentially conflicting quantitative and qualitative criteria can be taken into consideration, and interactions, dependencies, and feedback relationships between them can be investigated with hesitant fuzzy analytic network process (hesitant F-ANP).

## Figures and Tables

**Table 1:** COVID-19 intervention strategies and country examples.

A1	Quarantine/lockdown of patients and those suspected of infection	Policies to quarantine or shelter in place for at least 14 days. For example, “Hong Kong, a semiautonomous Chinese region, requires travelers from all countries to self-quarantine for 14 days”
A2	Internal border restrictions reducing the ability to move freely (transportation) within a country	Government policies which reduce the ability to move freely within a country. For example, in Peru as of March 15 2020, “officials are also restricting the movement of people across provinces”
A3	Social distancing	Government policies that limit physical contact between individuals to 1.5 meters or 6 feet. For example, in Germany, “a 1.5 meter (4.9 feet) distance should be kept at all times when in public”
A4	Health monitoring	Government policies that seek to monitor the health of individuals who are afflicted with or who are likely to be afflicted with the coronavirus. For example, “Taiwan CDC monitors all individuals who had traveled to Wuhan within 14 days and exhibited a fever or symptoms of upper respiratory tract infections”
A5	Public awareness campaigns	Efforts to disseminate and convey reliable information about COVID-19, including ways to prevent or mitigate the health effects of COVID-19. For example, on March 22, 2020, it was announced that “the Provincial Youth Council in Namibia carried out an intense public awareness campaign on methods of disease prevention, during which, young associates distributed pamphlets with statements about the pandemic and ways of prevention”
A6	Restriction of nonessential businesses	Government policies that restrict nonessential commercial activity. For example, in Serbia, “as of March 21, 2020, the following measures are in effect: supermarkets, gas stations, restaurants, post offices, banks and other service providers will be reducing their hours to observe the curfew, with some closing at 6 : 00 PM or earlier. Cafes, restaurants and shopping centers are closed, however food delivery is allowed”
A7	Restrictions of mass gatherings	Government policies that limit the number of people allowed to congregate in a place. For example, on March 16, 2020, in the United States, “the latest recommendation announced Monday by the federal government to promote social distancing and limit the transmission of the coronavirus is: no more than 10 people in one place”
A8	External border restrictions reducing the ability to exit or enter a country	Government policies which reduce the ability to access ports of entry to or exit from a country. For example, “Namibian government suspends inbound and outbound flights for 30 days”
A9	Closure of schools	Government policy that closes educational establishments in a country. For example, in Slovakia, as of March 12, 2020, “all schools and educational establishments will be shut down”
A10	Government policies that affect the country's health resources (materials and health worker)	Government policies which affect the material (e.g., medical equipment, number of hospitals for public health) or human (e.g., doctors, nurses) health resources of a country. For example, “Taiwan bans exports of face masks; ban extended to the end of April (2020)” or “Government approves plan to build 60 production lines to make an additional 6 million masks per day”
A11	Formation of new task units/bureaus and government policies changing administrative capacity to respond to the crisis	Government policy that changes the administrative capacity of a part of government to respond to the crisis. For example, on January 20, 2020, “Taiwan activated the Central Epidemic Command Center (CECC) which mobilizes government funds and military personnel to facilitate face mask production”
A12	Common health testing (independent of suspected infection)	Government policies which seek to sample large populations for coronavirus regardless of suspected likelihood of affliction with coronavirus.
A13	Curfew	Government policies that limit domestic freedom of movement to certain times of the day. For example, in Serbia, “as of March 21, 2020 the following measures are in effect: curfew for all residents with few exceptions from 8:00pm to 5:00am the next day”
A14	Restriction of nonessential government services	Government policy that restricts nonessential government services. For example, in Malaysia, from March 18, 2020, to March 31, 2020, “all government and private services except those involved in essential services such as water, electricity, power, telecommunications, postal, transportation, fuel, finance, banking, health, pharmacy, fire, port, airport, security, retail and food supply will also be closed”
A15	Declaration of emergency	The head of government declares a state of national emergency. For example, on March 15, 2020, in South Africa: “President Ramaphosa announces national state of disaster”

**Table 2:** Scale for the evaluation of importance of intervention strategy alternatives in hesitant F-AHP [53].

Linguistic terms	Triangular fuzzy number (TFN)
Absolutely strong (AS)	(2, 5/2, 3)
Very strong (VS)	(3/2, 2, 5/2)
Fairly strong (FS)	(1, 3/2, 2)
Slightly strong (SS)	(1, 1, 3/2)
Equal (E)	(1, 1, 1)
Slightly weak (SW)	(2/3, 1, 1)
Fairly weak (FW)	(1/2, 2/3, 1)
Very weak (VW)	(2/5, 1/2, 2/3)
Absolutely weak (AW)	(1/3, 2/5, 1/2)

**Table 3:** Pairwise comparison of importance of COVID-19 intervention strategies by 7 DMs.

	A1	A2	A3	A4	A5	A6	A7	A8	A9	A10	A11	A12	A13	A14	A15
A1	E	FS	FW	FS	FS	VS	E	FS	AW	E	E	AW	AW	FS	FW
	E	VS	VS	AS-VS	VS-FS	VS	SS	SS	E	E	SS	SW-FW	FW-VW	FW	VW
	E	AS	AS	AS	AS	AS	AS	AS	AS	AS	AS	AS	AS	AS	AS
	E	AS	FS	AS	FS-SS	FS	AS	AS	AS	AS-FS	FS	AS	FS-SS	FS	FS-SS
	E	VS	AS	AS	AS	VS	VS	SS	VS	SS	E	SS	FW	E	AW
	E	AS	AS	AS	AS	AS	AS	AS-VS	SS	E	SS	E	E	FS	E
	E	FS-SS	FW	FS	VS	AS	SS-E	E	FW	SW-FW	VS	FS	VS-FS	VS	AW

A2		E	SW	FS	FS	SW	SW	E	AW	SW	FW	AW	AW	FW	FW
		E	SW-E	E	SW	SW	FW	FW	VW	E	SW	VW	VW	VW	AW
		E	VS	VS	AS	E	E	E	E-SW	FW	SW-FW	VW	E-SW	E-SW	FW
		E	FS	SS	SS-VS	AS	FS	AS	AS	AS-FS	FS	AS	FS-SS	FS	FS
		E	SW	SW	SS	SW	SW	FW	SW	E	SS	SS	FW	SW	VW
		E	SW	FW	FW	FS	E-SW	E	E	E	SS	E	E	E	FW
		E	VS	FS	AS	VS-FS	SS	E-SW	FS	VS	FS	VS-FS	SS-E	FS	SS-E

A3			E	VS	AS	VS	AS	FS	E	FS	FS	E	E	FS	FS
			E	VS	VS-FS	FS	SS	SS	FW	SW	SW	AW	AW	SW	AW
			E	SW-FW	FS	E-SW	SW	SW-FW	SW-FW	FW	SW	FW	FW	FW	FW-VW
			E	FS-AS	SS-FS	AS	FS	FS-SS	SS	SS	SS	FW	AS	FS	FS
			E	SS	VS	E	E	SW	E	SS	SS	AS	SW	E	SW
			E	E	VS	FS	E	E	E	SS	FS	E	FW	FS	SW-FW
			E	VS	VS	VS	SS-E	FW	E-SW	VS	AS	FS	FS-SS	VS	SS-E

A4				E	E	SW	SW	FW	VW	FW	SW	VW	AW	SW	AW
				E	SS	SW	SW-E	FW	SW	SW	FW	AW	AW	VW	AW
				E	AS	AS	SS	SS-E	FS	E	E	E	SS-E	SS-E	E
				E	FS	FS	FS	FS-SS	FS	FS	SS	FS	SW	SW	SW
				E	SS	SW	SW	FW	SW	SS	E	VS	SW	SW	VW
				E	AS	AS	VS	E	E	SS	FS-SS	E	E	FS	E
				E	SS-E	SS-E	FW-VW	FW-AW	VW	SS	FS	SS	SW-FW	FS	AW

A5					E	E	FW	FW	AW	AW	VW	AW	AW	SW	AW
					E	E	SW	SW	FW	FW	FW	AW	AW	VW	AW
					E	SW	FW	SW	SW	SW-FW	SW	FW-VW	FW-VW	FW	FW-VW
					E	FS	SS	FS	FS	SS	SS	SW	SS	FS	SS
					E	SW	SW	AW	SW	SS	SS	FS	SW	SW	AW
					E	FW	FW	FW	VW	FW	E	VW	AW	FW	VW
					E	E-SW	VW	AW	VW	SS-E	FS	VS	SS	SW	AW

A6						E	E	E	AW	FW	FW	AW	AW	E	AW
						E	SW-E	E	FW	SW-E	VW	AW	AW	FW	AW
						E	E-SW	FS	SS	E	E	E	FW-VW	E	FW-VW
						E	SW	FW	AW-VW	SW	SS	VW	FW	E	FW
						E	SW	AW	SW	SS	SS	FS	SW	SW	AW
						E	FW	VW	FW	FW	SW	FW	VW	E	FW-VW
						E	SW-FW	VW	FW	E-SW	E	VW	VW	SW	AW

A7							E	FS	SW	FS	E	VW	AW	FS	AW
							E	SW-E	E	SW	FW	VW	AW	SW	AW
							E	VS	FS-SS	E	E	E	FW	E	VW
							E	FW	E	SW	SS	SW	FW	E	FW
							E	SW	E	SS	SW	FS	FW	E	VW
							E	E	E	SW	SS	SW	SW	E	FW
							E	E-SW	E	VS	AS-VS	VS	VS	VS	SS-E
A8								E	FW	SW	FW	AW	AW	VW	AW
								E	E	SS	SS	VW	VW	E	VW
								E	E	SW	SW	FW	FW-VW	SW	VW
								E	SS	FS	SS	SW	E	E	E
								E	AS	FS	FS	AS	VS	AS	SS
								E	E	SS	SS-FS	FW	SW	E	SW
								E	AS-VS	VS	AS	VS	FS	AS-VS	SS-E

A9									E	VS	VS	SW	AW	VS	AW
									E	VS	VS	VW	SW	E	FW
									E	FW-SW	FW-SW	VW	VW	SW-E	VW
									E	FS	FS	E	SS	FS	E
									E	SS	SW	FS	SW	E	VW
									E	SW	SS	SW	E	E	SW
									E	VS	AS	FS	FS-SS	FS	SW-FW

A10										E	E	AW	AW	FW	AW
										E	E	FW	VW	VW	AW
										E	E	E	E	SS-E	E
										E	E	FW	FS	FS	E
										E	SW	SS	SW	SW	SW
										E	E	SW	SW	SS	SW
										E	FS	FW	FW-VW	FW	AW

A11											E	AW	AW	FW	AW
											E	FW	FW	VW	AW
											E	E	E	E	E
											E	E	SS	FS	E
											E	SS	SW	SW	FW
											E	FW	SW	E	FW
											E	VW	FW-VW	FW	AW

A12												E	AS	AS	AS
												E	VS	VS	E
												E	E	SS-E	E
												E	E	AS	AS
												E	FW	FW	AW
												E	E	SS	E
												E	SW-FW	FS-SS	FW

A13													E	AS	AW
													E	FS	FW
													E	FS	E
													E	AS	E
													E	VS	SW
													E	FS	FW
													E	AS-VS	E-SW

A14														E	AW
														E	FW
														E	SW
														E	FW
														E	SW
														E	SW-FW
														E	VW-AW

A15															E
															E
															E
															E
															E
															E

**Table 4:** The fuzzy evaluation matrix for the intervention strategy alternatives ($\tilde{X}$).

	A1	A2	A3	A4	A5
A1	(1.000, 1.000, 1.000)	(1.571, 2.000, 2.571)	(1.357, 1.763, 2.214)	(1.643, 2.143, 2.714)	(1.500, 1.929, 2.571)
A2	(0.399, 0.506, 0.691)	(1.000, 1.000, 1.000)	(0.954, 1.357, 1.571)	(0.953, 1.239, 1.571)	(1.167, 1.586, 2.000)
A3	(0.556, 0.767, 1.024)	(0.757, 0.810, 1.191)	(1.000, 1.000, 1.000)	(1.143, 1.586, 2.000)	(1.357, 1.786, 2.429)
A4	(0.379, 0.477, 0.667)	(0.724, 0.906, 1.167)	(0.601, 0.735, 1.001)	(1.000, 1.000, 1.000)	(1.286, 1.500, 1.929)
A5	(0.399, 0.506, 0.739)	(0.604, 0.846, 1.071)	(0.419, 0.534, 0.787)	(0.595, 0.781, 0.857)	(1.000, 1.000, 1.000)
A6	(0.384, 0.481, 0.644)	(0.747, 0.796, 1.143)	(0.590, 0.677, 0.906)	(0.690, 0.781, 1.071)	(0.929, 1.024, 1.357)
A7	(0.532, 0.671, 0.739)	(0.881, 1.024, 1.357)	(0.738, 0.867, 1.000)	(0.796, 0.953, 1.381)	(1.024, 1.357, 1.786)
A8	(0.548, 0.696, 0.810)	(0.904, 1.057, 1.286)	(0.810, 0.977, 1.357)	(0.881, 1.371, 1.714)	(1.214, 1.524, 2.000)
A9	(0.819, 1.043, 1.239)	(1.047, 1.224, 1.571)	(0.953, 1.071, 1.357)	(1.000, 1.191, 1.571)	(1.214, 1.524, 2.000)
A10	(0.762, 0.863, 1.071)	(0.819, 0.949, 1.167)	(0.701, 0.953, 1.167)	(0.787, 1.024, 1.214)	(1.001, 1.357, 1.714)
A11	(0.653, 0.796, 0.881)	(0.763, 0.977, 1.357)	(0.667, 0.820, 1.071)	(0.810, 0.977, 1.214)	(0.906, 1.167, 1.429)
A12	(0.833, 0.996, 1.286)	(1.057, 1.343, 1.643)	(0.976, 1.224, 1.500)	(1.010, 1.239, 1.453)	(1.200, 1.524, 2.024)
A13	(0.890, 1.153, 1.571)	(1.095, 1.381, 1.714)	(0.976, 1.224, 1.571)	(1.238, 1.429, 1.857)	(1.334, 1.714, 2.143)
A14	(0.604, 0.773, 1.024)	(0.929, 1.120, 1.500)	(0.700, 0.859, 1.167)	(0.881, 1.049, 1.429)	(1.000, 1.239, 1.714)
A15	(1.190, 1.510, 1.857)	(1.095, 1.524, 1.929)	(0.952, 1.191, 1.714)	(1.500, 1.786, 2.143)	(1.596, 2.071, 2.571)

	A6	A7	A8	A9	A10
A1	(1.643, 2.143, 2.643)	(1.500, 1.786, 2.214)	(1.357, 1.643, 2.143)	(1.190, 1.439, 1.786)	(1.071, 1.371, 1.643)
A2	(1.001, 1.357, 1.643)	(0.787, 1.024, 1.214)	(0.952, 1.120, 1.286)	(0.867, 1.129, 1.310)	(0.953, 1.300, 1.500)
A3	(1.238, 1.643, 2.000)	(1.096, 1.286, 1.571)	(0.810, 0.977, 1.357)	(0.810, 0.906, 1.071)	(0.953, 1.167, 1.571)
A4	(1.144, 1.500, 1.786)	(0.844, 1.143, 1.429)	(0.691, 0.808, 1.214)	(0.734, 1.000, 1.191)	(0.881, 1.024, 1.357)
A5	(0.787, 1.024, 1.143)	(0.606, 0.787, 1.024)	(0.571, 0.806, 1.000)	(0.567, 0.796, 0.977)	(0.690, 0.773, 1.143)
A6	(1.000, 1.000, 1.000)	(0.668, 0.906, 1.000)	(0.661, 0.796, 0.977)	(0.548, 0.687, 0.952)	(0.715, 0.906, 1.071)
A7	(1.000, 1.071, 1.571)	(1.000, 1.000, 1.000)	(0.858, 1.167, 1.357)	(0.953, 1.000, 1.143)	(0.930, 1.214, 1.429)
A8	(1.214, 1.524, 1.857)	(0.843, 0.953, 1.310)	(1.000, 1.000, 1.000)	(1.143, 1.310, 1.643)	(0.977, 1.286, 1.643)
A9	(1.167, 1.571, 2.071)	(0.929, 0.953, 1.071)	(0.762, 0.900, 1.024)	(1.000, 1.000, 1.000)	(1.096, 1.452, 1.857)
A10	(0.953, 1.143, 1.500)	(0.796, 0.881, 1.167)	(0.677, 0.834, 1.096)	(0.624, 0.739, 1.073)	(1.000, 1.000, 1.000)
A11	(0.977, 1.214, 1.429)	(0.811, 0.986, 1.167)	(0.667, 0.891, 1.143)	(0.614, 0.724, 1.049)	(0.929, 0.953, 1.071)
A12	(1.357, 1.739, 2.143)	(0.986, 1.167, 1.524)	(1.033, 1.343, 1.739)	(1.000, 1.191, 1.571)	(1.096, 1.429, 1.786)
A13	(1.429, 1.857, 2.429)	(1.200, 1.571, 2.024)	(1.057, 1.310, 1.739)	(1.096, 1.310, 1.643)	(1.143, 1.381, 1.857)
A14	(1.000, 1.071, 1.286)	(0.843, 0.881, 1.024)	(0.881, 0.971, 1.167)	(0.771, 0.834, 1.024)	(0.905, 1.239, 1.571)
A15	(1.571, 2.071, 2.714)	(1.381, 1.857, 2.286)	(1.191, 1.500, 1.786)	(1.286, 1.571, 2.071)	(1.429, 1.643, 2.000)

	A11	A12	A13	A14	A15
A1	(1.214, 1.429, 1.786)	(1.119, 1.367, 1.714)	(0.890, 1.081, 1.571)	(1.143, 1.524, 1.929)	(0.794, 0.924, 1.239)
A2	(0.810, 1.048, 1.429)	(0.876, 1.057, 1.406)	(0.700, 0.796, 1.096)	(0.748, 1.024, 1.239)	(0.604, 0.773, 1.096)
A3	(1.049, 1.357, 1.714)	(0.904, 1.106, 1.357)	(0.857, 1.034, 1.357)	(0.953, 1.310, 1.643)	(0.700, 0.939, 1.286)
A4	(0.881, 1.024, 1.357)	(0.890, 1.057, 1.310)	(0.643, 0.781, 0.929)	(0.773, 1.071, 1.310)	(0.580, 0.671, 0.739)
A5	(0.796, 0.953, 1.239)	(0.661, 0.900, 1.167)	(0.580, 0.671, 0.929)	(0.630, 0.906, 1.096)	(0.446, 0.514, 0.739)
A6	(0.796, 0.881, 1.096)	(0.566, 0.710, 0.906)	(0.433, 0.567, 0.763)	(0.834, 0.953, 1.000)	(0.374, 0.467, 0.714)
A7	(0.953, 1.096, 1.429)	(0.806, 1.071, 1.263)	(0.619, 0.830, 1.071)	(1.024, 1.214, 1.357)	(0.494, 0.591, 0.834)
A8	(1.024, 1.239, 1.714)	(0.843, 1.106, 1.381)	(0.757, 0.986, 1.239)	(1.081, 1.286, 1.524)	(0.686, 0.771, 0.977)
A9	(1.167, 1.524, 1.929)	(0.734, 1.000, 1.191)	(0.724, 0.843, 1.096)	(1.024, 1.286, 1.500)	(0.543, 0.677, 0.834)
A10	(0.953, 1.071, 1.143)	(0.643, 0.773, 1.000)	(0.639, 0.843, 1.024)	(0.724, 0.906, 1.239)	(0.619, 0.743, 0.786)
A11	(1.000, 1.000, 1.000)	(0.676, 0.749, 0.953)	(0.653, 0.796, 1.000)	(0.724, 0.906, 1.096)	(0.570, 0.649, 0.786)
A12	(1.167, 1.500, 1.786)	(1.000, 1.000, 1.000)	(1.071, 1.263, 1.500)	(1.286, 1.524, 2.071)	(1.119, 1.296, 1.500)
A13	(1.096, 1.357, 1.786)	(0.819, 0.914, 1.167)	(1.000, 1.000, 1.000)	(1.429, 1.929, 2.500)	(0.667, 0.820, 0.929)
A14	(1.000, 1.239, 1.571)	(0.557, 0.781, 0.953)	(0.413, 0.530, 0.762)	(1.000, 1.000, 1.000)	(0.501, 0.687, 0.881)
A15	(1.429, 1.786, 2.143)	(0.951, 1.114, 1.286)	(1.143, 1.357, 1.714)	(1.214, 1.500, 2.143)	(1.000, 1.000, 1.000)

**Table 5:** *X* and intervention strategy alternative scores (*w*).

	A1	A2	A3	A4	A5	A6	A7	A8	A9	A10	A11	A12	A13	A14	A15	*w*
A1	1.000	2.024	1.770	2.155	1.964	2.143	1.810	1.679	1.455	1.367	1.452	1.384	1.131	1.528	0.955	0.0936
A2	0.519	1.000	1.326	1.246	1.585	1.345	1.016	1.120	1.115	1.275	1.072	1.085	0.830	1.014	0.799	0.0644
A3	0.775	0.865	1.000	1.581	1.821	1.635	1.302	1.013	0.917	1.199	1.365	1.114	1.059	1.306	0.957	0.0704
A4	0.492	0.919	0.757	1.000	1.536	1.488	1.141	0.856	0.988	1.056	1.056	1.071	0.783	1.061	0.667	0.0580
A5	0.527	0.843	0.557	0.763	1.000	1.004	0.796	0.799	0.788	0.821	0.974	0.905	0.699	0.891	0.540	0.0471
A6	0.492	0.845	0.701	0.814	1.064	1.000	0.882	0.804	0.708	0.902	0.903	0.719	0.577	0.941	0.493	0.0464
A7	0.659	1.056	0.868	0.998	1.373	1.143	1.000	1.147	1.016	1.203	1.127	1.059	0.835	1.206	0.616	0.0604
A8	0.690	1.070	1.013	1.347	1.552	1.528	0.994	1.000	1.338	1.294	1.282	1.108	0.990	1.291	0.791	0.0680
A9	1.038	1.253	1.099	1.223	1.552	1.587	0.969	0.898	1.000	1.460	1.532	0.988	0.865	1.278	0.681	0.0685
A10	0.881	0.964	0.947	1.016	1.357	1.171	0.915	0.852	0.775	1.000	1.064	0.789	0.839	0.931	0.729	0.0566
A11	0.786	1.005	0.836	0.989	1.167	1.210	0.987	0.896	0.760	0.969	1.000	0.770	0.806	0.907	0.658	0.0545
A12	1.017	1.345	1.229	1.236	1.554	1.742	1.196	1.357	1.223	1.433	1.492	1.000	1.270	1.576	1.300	0.0799
A13	1.179	1.389	1.241	1.468	1.722	1.881	1.585	1.339	1.330	1.421	1.385	0.940	1.000	1.940	0.813	0.0811
A14	0.787	1.151	0.884	1.084	1.278	1.095	0.899	0.989	0.855	1.239	1.254	0.773	0.549	1.000	0.688	0.0572
A15	1.515	1.520	1.239	1.798	2.075	2.095	1.849	1.496	1.607	1.667	1.786	1.116	1.381	1.560	1.000	0.0938

## Data Availability

All the data used to support the findings of this study are included within the article.
